# Conjunctival retraction with deposits after trabeculectomy

**DOI:** 10.1016/j.ajoc.2025.102466

**Published:** 2025-11-07

**Authors:** Bei Zeng, Zidong Chen, Minbin Yu

**Affiliations:** State Key Laboratory of Ophthalmology, Zhongshan Ophthalmic Center, Sun Yat-sen University, Guangdong Provincial Key Laboratory of Ophthalmology and Visual Science, Guangzhou, China

## Abstract

**Purpose:**

To describe a case of late-onset conjunctival flap retraction after fornix-based trabeculectomy, presenting with chronic subconjunctival accumulation of yellow crystalline material, which responded to antifungal and broad-spectrum antibiotic therapy.

**Observation:**

A 26-year-old female patient with a history of congenital glaucoma underwent combined trabeculotomy and fornix-based trabeculectomy in the left eye at 4 months of age. Over the years, the patient experienced recurrent episodes of redness, responding to topical antibiotics. Two months prior to presentation, the patient developed persistent ocular redness and yellow discharge in the left eye. Examination revealed conjunctival hyperemia, conjunctival flap retraction with sub-conjunctival yellow crystalline deposits, and suture debris. Microbiological testing of the conjunctival sac contents identified fungal (*Cladosporium* spp.) and opportunistic bacterial (*Micrococcus luteus*) infections. The patient improved significantly after removal of the debris and administration of topical antifungal and broad-spectrum antibiotics.

**Conclusions and importance:**

Complications of trabeculectomy-related conjunctival flap have most commonly been reported as conjunctival flap fibrosis and infection. We present a rare case of conjunctival flap retraction leading to the formation of a subconjunctival cavity with crystalline deposits, complicated by chronic fungal infection.

## 1 Introduction

Glaucoma remains the leading cause of irreversible blindness worldwide. In infants with congenital glaucoma, surgery is considered the first-line treatment, most commonly trabeculotomy or trabeculectomy.^1^^,^^2^ Reported complications after trabeculectomy include conjunctival flap fibrosis, blebitis, and choroidal detachment. Among these, conjunctival flap-related infections are of particular concern, as they may progress to endophthalmitis and pose a severe threat to vision.^3^

The Collaborative Bleb-Related Infection Incidence and Treatment Study reported a 2.2 % incidence of conjunctival flap infection within five years. Younger age and a history of conjunctival flap leakage have been identified as risk factors for bleb-related infection.^4^ Fornix-based conjunctival flap is generally flatter and more diffuse, whereas limbus-based conjunctival flap is more likely to develop an elevated, cystic configuration that is prone to encapsulation.^5^ Here, we describe a rare case of late-onset chronic conjunctival flap infection characterized by crystalline deposits within retracted conjunctival flap capsule following a fornix-based trabeculectomy.

## 2 Case report

**Fig. 1:**
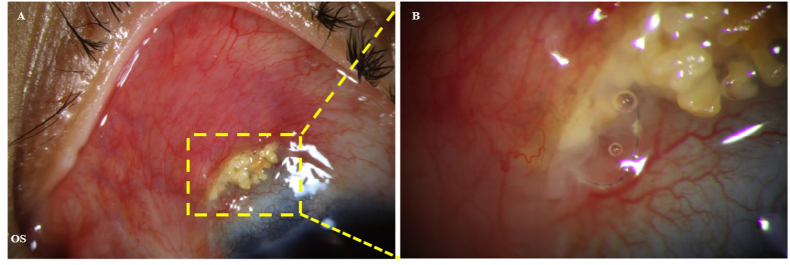
Photograph of the Filtering conjunctival flap in the Left Eye. A: Shows superior conjunctival hyperemia with retraction of the conjunctival flap margin. Yellow crystalline deposits and suture debris are observed within the subconjunctival pocket, highlighted by dashed yellow lines. Figure B: Provides a magnified view of Figure A. (For interpretation of the references to colour in this figure legend, the reader is referred to the Web version of this article.)

**Fig. 2:**
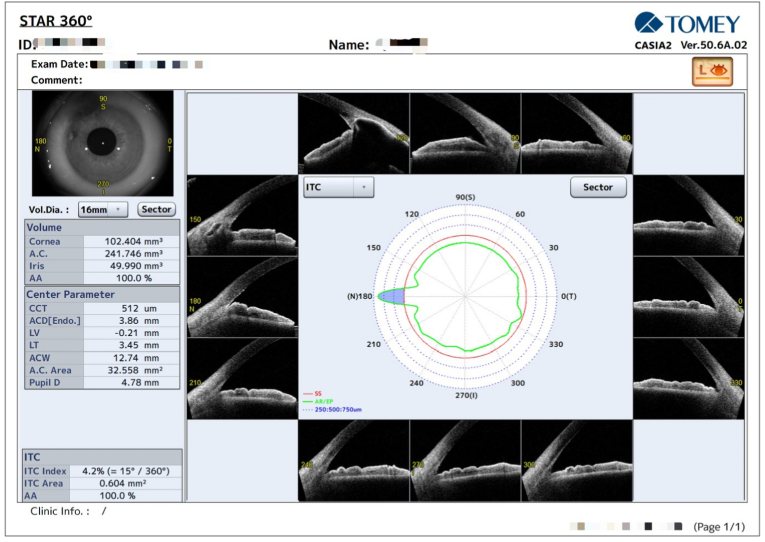
Anterior Segment Optical Coherence Tomography (AS-OCT, CASIA 2, Tomey) Image of the Left Eye. Iridotrabecular contact (ITC) was minimal, with an ITC index of 4.2 % (15°/360°), localized to the nasal quadrant (180°), as indicated by the blue sector on the ITC polar map. (For interpretation of the references to colour in this figure legend, the reader is referred to the Web version of this article.)

A 26-year-old woman with congenital glaucoma had undergone trabeculotomy combined with fornix-based trabeculectomy in the left at 4 months of age. She reported recurrent episodes of conjunctival hyperemia in the left eye over the past several years, each resolving temporarily with topical antibiotic therapy. Two months before presentation, she developed worsening redness associated with yellow discharge, which did not improve with over-the-counter antibiotic drops. On examination, superior conjunctival hyperemia and conjunctival flap margin retraction were noted in the left eye (Fig. 1). Yellow crystalline deposits and residual suture material were observed within the subconjunctival pocket. The patient had tenderness on palpation, and copious yellow crystalline discharge was expressed. Best-corrected visual acuity was 20/40, and intraocular pressure was 13 mmHg without topical glaucoma medications. Anterior segment evaluation revealed iris incarceration at the superior nasal limbus with localized peripheral anterior synechiae, while the remaining angle was open (Fig. 2). The central anterior chamber was deep, and the crystalline lens was clear but showed scattered surface pigment granules. Fundus examination showed a cup-to-disc ratio of 0.6 in the left eye. Given persistent symptoms despite topical ofloxacin use, fungal infection was suspected. A sample of the discharge from the subconjunctival pocket was collected for microbiological analysis, and the space was thoroughly irrigated. Culture of the specimen yielded *Cladosporium* spp. and *Micrococcus luteus*. Empirical therapy with a broad-spectrum antibiotic (0.3 % tobramycin ophthalmic solution) and antifungal agents (1 % voriconazole ophthalmic solution and 0.5 % fluconazole ophthalmic ointment) was initiated. Based on susceptibility testing, the regimen was subsequently adjusted to 0.3 % tobramycin and 1 % voriconazole, which were administered for three weeks and resulted in marked clinical improvement.

Review of prior records revealed that one year postoperatively, iris incarceration beneath the conjunctiva accompanied by superior peripheral anterior synechiae was documented. Intraocular pressure remained within physiological limits without topical glaucoma medications. The patient was advised to undergo regular surveillance, during which intraocular pressure measurements consistently remained stable without topical glaucoma medications. Fifteen years postoperatively, conjunctival flap retraction and eyelash entrapment within the conjunctival flap capsule were noted. These chronic structural alterations might cause a predisposition for persistent, low-grade conjunctival flap infection without evidence of intraocular extension. A comprehensive review of the patient's medical history and records revealed no evidence of systemic disease or immunocompromise, and no clinical features suggestive of immunosuppression were observed.

## 3 Discussion

We report a rare case of conjunctival flap margin retraction following fornix-based trabeculectomy, associated with the formation of yellow subconjunctival crystalline deposits. Clinical symptoms improved markedly after the subconjunctival crystalline deposits were thoroughly removed, combined with topical antifungal and broad-spectrum antibiotic therapy.

The subconjunctival deposits resembled conjunctival concretions, which are yellow crystalline accumulations typically observed on the palpebral conjunctiva. Histologically, conjunctival concretions consist of degenerative epithelial cells, mucus from conjunctival glands, and inflammatory cells, particularly eosinophilic amorphous material that stains positively with periodic acid–Schiff. Calcification is usually minimal.^6^ The formation of conjunctival concretions is closely associated with surface irregularities, such as those seen in patients with trachomatous scarring, often accumulating in scarred crypts.^7^ Fungal hyphae have also been identified in some cases, suggesting a link between chronic fungal colonization and concretion formation.^8^ *Cladosporium* spp. are fungi that are ubiquitous in various environmental settings and constitute a segment of the normal human skin microbiota.^9^ *Micrococcus luteus* is a Gram-positive bacterium. Research has detected the presence of *Micrococcus luteus* in conjunctival secretion cultures following the use of broad-spectrum antibiotics prior to ophthalmic surgery, suggesting its capacity to induce opportunistic infections and highlighting the need for careful consideration.^10^^,^^11^ In this patient, superior conjunctival flap margin retraction created a potential subconjunctival cavity. Chronic entrapment of cilia and prolonged subclinical infection likely expanded this place. Under sustained irritation from fungal organisms and foreign bodies, yellow crystalline deposits subsequently formed within the conjunctival flap capsule.

Conjunctival flap scarring is reported to be less frequent in fornix-based conjunctival flap compared to limbus-based conjunctival flap.^5^ In this case, retraction of the conjunctival margin created a subconjunctival cavity. Early postoperative iris incarceration beneath the conjunctiva likely obstructed aqueous outflow, isolating the conjunctival flap from the anterior chamber. Pigmented tissue was visible on the scleral flap surface. As a result, infection was confined to the conjunctival surface without intraocular involvement. Prompt removal of discharge and suture debris, along with appropriate antifungal and broad-spectrum topical antibiotics, resulted in significant symptomatic improvement. A thorough review of the patient's medical history and records revealed no evidence of immunocompromise or immune-related disease, and no clinical features of recurrent systemic infections were observed. Nevertheless, the entrapped eyelashes were not cultured, and the exact source of the infection could not be determined, which should be acknowledged as a limitation of this report.

## 4 Conclusion

In summary, this case illustrates that even decades after fornix-based trabeculectomy, retraction of the conjunctival flap margin can result in the formation of a subconjunctival pocket prone to chronic infection. Under prolonged fungal and foreign body irritation, yellow crystalline deposits may develop. Timely recognition and treatment with antifungal and broad-spectrum antibiotics can achieve good outcomes. We recommend that long-term follow-up of fornix-based trabeculectomy include careful monitoring for conjunctival flap retraction and late-onset conjunctival flap-related complications.

## CRediT authorship contribution statement

**Bei Zeng:** Writing – original draft, Investigation, Data curation. **Zidong Chen:** Writing – review & editing. **Minbin Yu:** Writing – review & editing, Supervision, Funding acquisition, Conceptualization.

## Patient consent

Written informed consent was obtained from the patient.

## Authorship

All authors attest that they meet the current ICMJE criteria for Authorship.

## Funding resource

This work was supported by Major-Outcome Cultivation Program, Zhongshan Ophthalmic Center of Excellence (Grant No. 304020115).

## Declaration of competing interest

Bei Zeng, Zidong Chen and Minbin Yu have no conflicts of interest to disclose.
